# Supplementary material for: A comparative study of RF heating of deep brain stimulation devices in vertical vs. horizontal MRI systems
Source: PLoS One. 2022 Dec 9;17(12):e0278187. doi: 10.1371/journal.pone.0278187 (PMC9733854; doi:10.1371/journal.pone.0278187)
Supplement: S1 File — (PDF) [file pone.0278187.s001.pdf]

Supplementary Material

S1 Table. Examples of routine MRI sequence parameters and scanner-reported $B_1^+{}_{rms}$ values for different imaging protocols. Data is taken from a clinical 1.5 T scanner at Northwestern Memorial Hospital.

**1.5 T Siemens Avanto**					
**Head Landmark**					
**Protocol**	**TE (ms)**	**TR (ms)**	**TA (min)**	**FA (°)**	**B_1^+ (µT)**
SE T1 SAG	10	450	2:09	90	3.54
AX FLAIR	86	9000	3:02	150	2
AX T1 SE	12	450	2:26	150	3.04
AX T2 TSE	94	3800	2:22	150	3.71
**Chest Landmark**					
**Protocol**	**TE (ms)**	**TR (ms)**	**TA (min)**	**FA (°)**	**B1+ (µT)**
COR TRUFI	1.21	491.33	0:20	64	4.01
AX TRUFI	1.18	328.38	0:13	64	3.99
AX VIBE	2.39, 4.77	6.9	0:20	10	1.49
TRUFI CINE 2C	1.16	38.22	0:06	60	3.98
TRUFI CINE SA	1.16	40.96	1:00	60	3.99
**Abdomen Landmark**					
**Protocol**	**TE (ms)**	**TR (ms)**	**TA (min)**	**FA (°)**	**B1+ (µT)**
T2 HASTE COR MBH	91	1400	0:48	180	4.2
T2 HASTE FS TRA MBH	94	1400	0:05	160	3.1

T2 BLADE FS TRA	91	2200	2:35	160	3.35
T2 TSE FS TRA MBH	86	4780	0:54	160	3.77

Simulations were performed with the anthropomorphic phantom—consisting of brain tissue-mimicking material, the skull, and saline—used during RF heating experiments and a heterogeneous human body model from ANSYS consisting of 32 tissue classes (Fig. S1 and S2). Simulations were performed with both vendor-specific transmit body coils for the 1.5 T Aera scanner and the 1.2 T Oasis scanner. These simulations were conducted in the absence of implanted DBS systems. The E-field distributions at three planes were calculated from simulations (Fig. S3 and S4). In all simulations, the input voltage was adjusted to produce a mean B_1^+ of 4 μT on an axial plane passing through the center of head. The E-field distributions were similar between the heterogeneous body model and our anthropomorphic phantom, demonstrating that the anthropomorphic phantom is a good representation of *in vivo* scenarios.

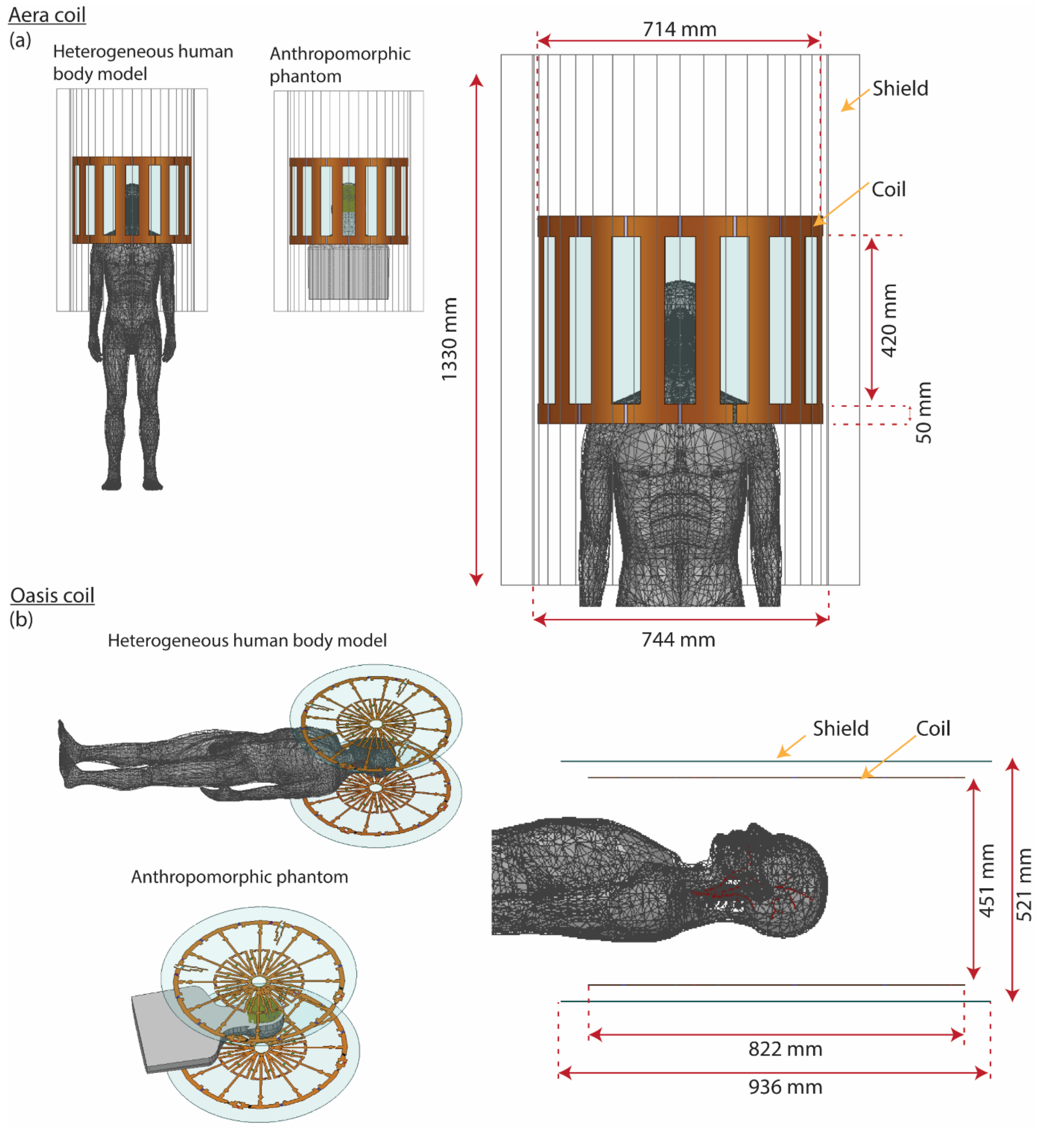

S1 Fig. (a) Simulation setup of the heterogeneous human body model and the anthropomorphic phantom in the 1.5 T Aera horizontal coil. The coil and shield dimensions are also provided. (b) Simulation setup of the heterogeneous human body model and the anthropomorphic phantom in the 1.2 T Oasis vertical coil. The coil and shield dimensions are also provided.

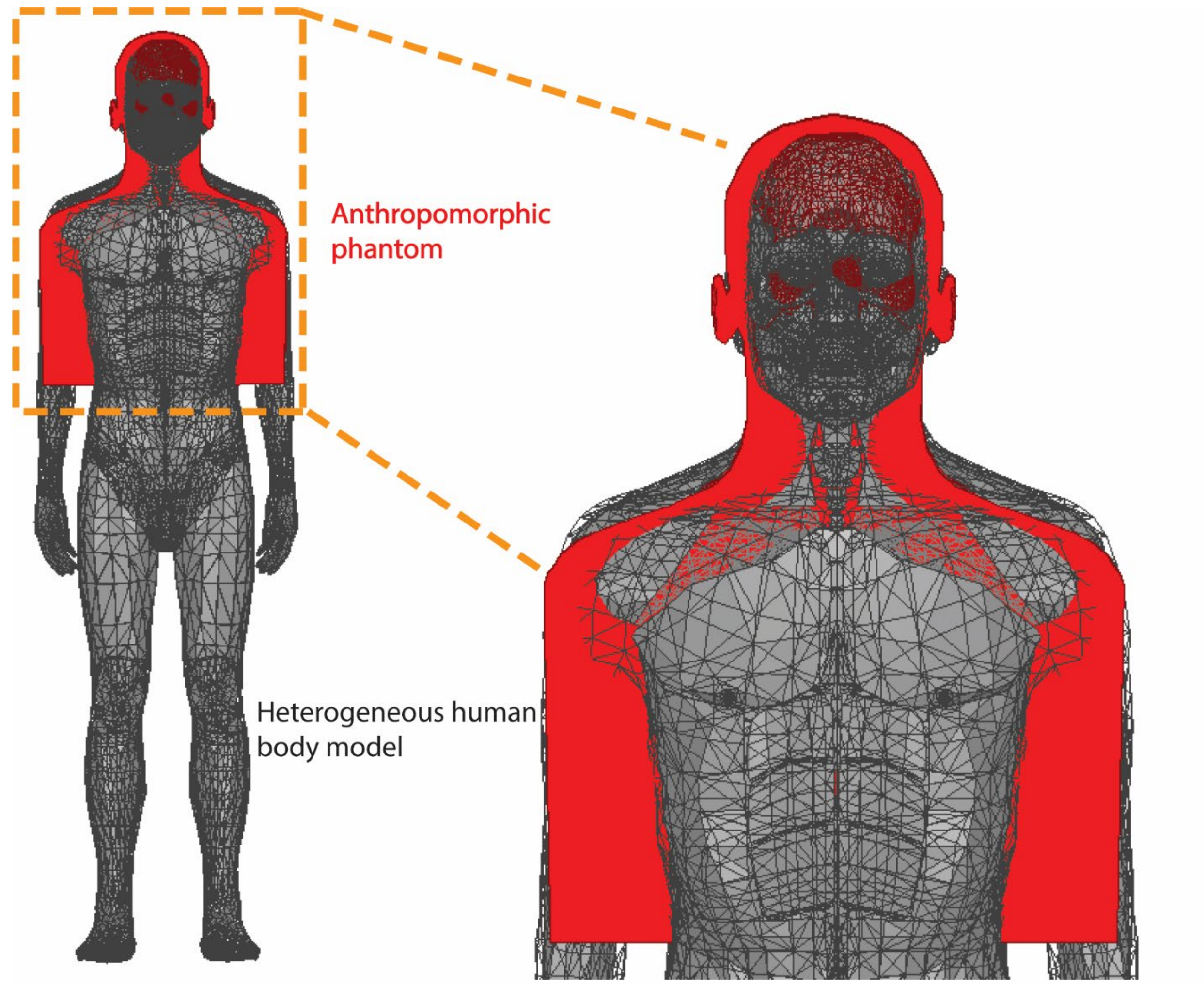

S2 Fig. Superposition of the anthropomorphic phantom used in experiments and the heterogeneous human body model from ANSYS for size comparison.

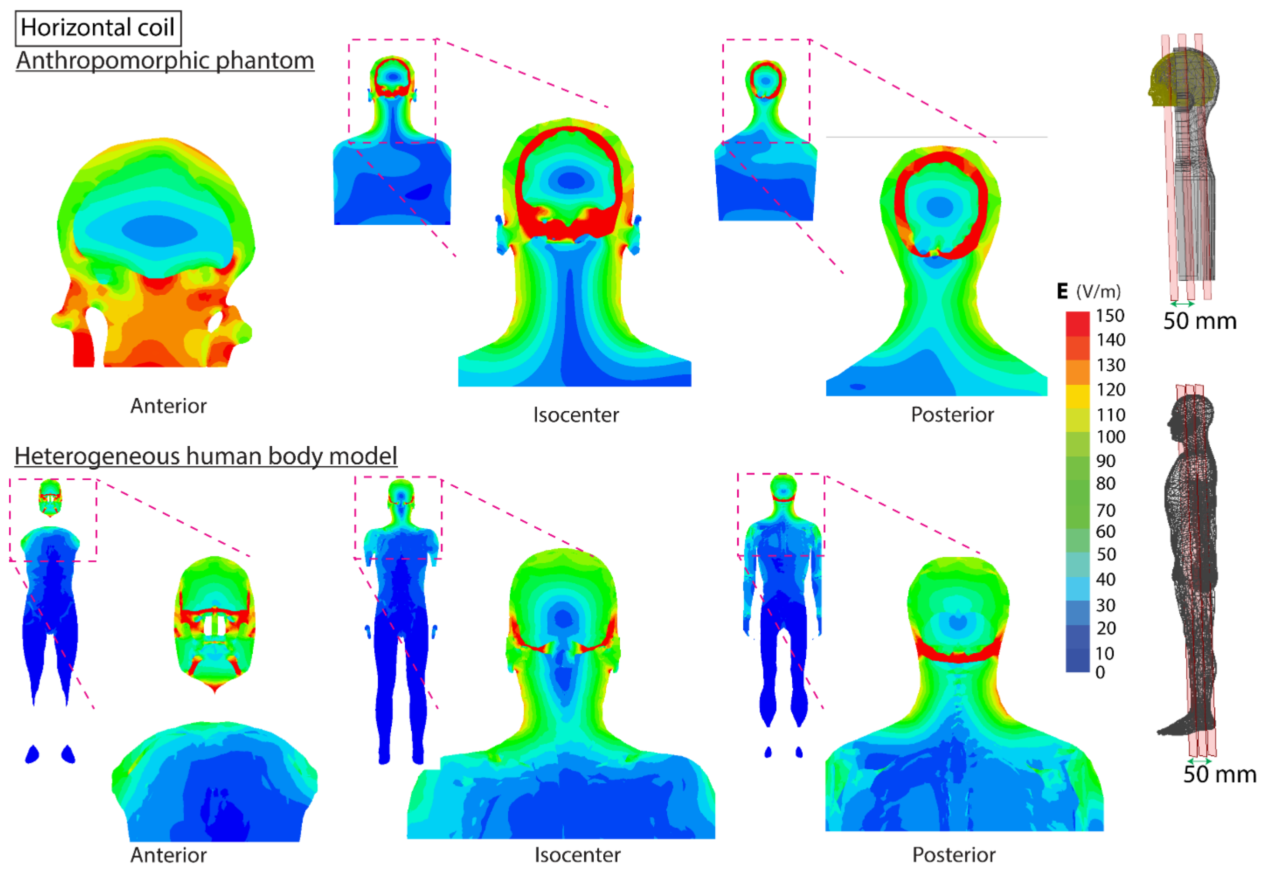

S3 Fig. Electric field distributions on coronal planes in the anthropomorphic phantom (top row) and heterogeneous human body model (bottom row) in the 1.5 T Aera horizontal coil.

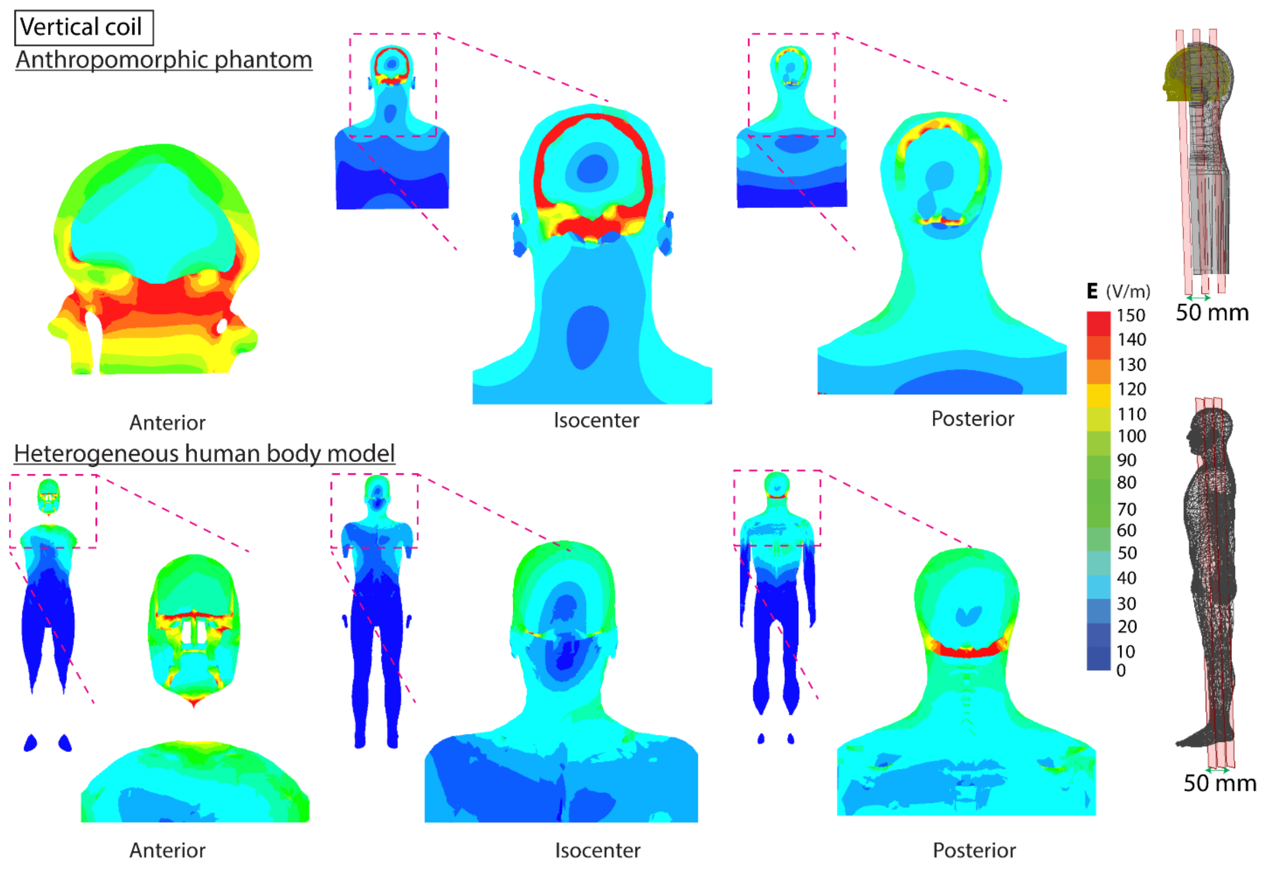

S4 Fig. Electric field distributions on coronal planes in the anthropomorphic phantom (top row) and heterogeneous human body model (bottom row) in the 1.2 T Oasis vertical coil.
