# Supplementary material for: A comparative study of RF heating of deep brain stimulation devices in vertical vs. horizontal MRI systems
Source: PLoS One. 2022 Dec 9;17(12):e0278187. doi: 10.1371/journal.pone.0278187 (PMC9733854; doi:10.1371/journal.pone.0278187)
Supplement: Attachment — Submitted filename: Response to Reviewers.docx [file pone.0278187.s003.docx]

**Point-by-point answer to reviewer’s comments**

We sincerely thank the reviewers for their thorough perusal of our work and for their thoughtful comments. We performed additional experiments and simulations guided by the reviewers’ suggestions which are explained here in detail and reflected in the revised manuscript. We believe that our work has been improved thanks to the reviewers’ constructive feedback and hope that it is deemed appropriate for publication in the prestigious journal of PLOS ONE.

Reviewer #1: This is a follow-up study of RF induced heating of DBS leads. The authors have previously published differences in RF induced heating of DBS leads between a horizontal, closed-bore MRI system and a vertical, open-bore system in two MRM publications (Ref 11 and 12). The current work extends the previous studies with further fiber-optic measurements of temperature rise around a commercial DBS device implanted in an anthropomorphic phantom following 30 patient-derived configurations. In addition, the electric length of the lead was varied to explore transferability of the results to leads from other manufacturers.

The study is methodologically sound and of principle interest for the scientific and medical community.

However, I do have a few comments for the authors:

Introduction, page 4: The authors state that in the previous simulation studies “simplified DBS lead models” were used, and also the term “proof-of-concept studies” implicates that the previous work may be less comprehensive than the current one. However, when I looked at both MRM publications, I actually found the opposite. Especially in Ref 12 a total of 90 lead models were assessed compared to 30 in this work, and the authors already performed experimental measurements of temperature rise for 4 trajectories and three configurations. Maybe you could rephrase that part of the introduction, and also clearly describe whether the 30 assessed configurations here are a subset of the previous 90 configurations, or a totally new set that has not been explored before? If so, why was a new set needed? What is the difference between the old and new set? Since temperature measurements have been performed before, the first question for this paper has already been answered with the previous 12 test cases compared to an additional set of 30 in this paper. What is the added value?

**Answer:** We thank the reviewer for the comment and apologize if our explanation was not clear.

First, note that from the 90 simulated trajectories in Ref 12, only 12 cases were “full DBS systems”, that is, DBS systems in which all three components of the device (i.e., 40-cm lead, 50-cm extension, and the implantable pulse generator) were present. The remainder of the cases were “lead only” systems with only a simplified model of the 40-cm lead. To give a brief background on “lead-only” vs “full” DBS systems, DBS surgery is usually performed in two stages. First, the tip of the lead is implanted in the target (e.g., subthalamic nucleus) and the extracranial portion of the lead is placed under the skin and the patient is sent home to recover. MRI at this stage is useful for lead localization and target verification. A second surgery takes place ~2-3 weeks later, in which an extension cable is connected to the lead and routed subcutaneously along the neck toward an IPG implanted in the chest. Thus, the majority of MRI exams on DBS patients are performed with full DBS systems which are the subjects of the present work.

Also, note that from the 12 experiments in Ref 12, **only 4 were done with a real DBS device** (the rest of experiments used straight wires, posing the same problem as simulations with simplified models). Moreover, even experiments with a real DBS device were not based on realistic patient data (i.e., trajectories were artificially made, see Figure 6 of Ref 12), which raised the question whether the observed result is valid in a real patient population. Finally, the DBS device itself was from a different manufacturer (Medtonic, as opposed to Abbott in the present work).

The 30 trajectories included in this work are 1) extracted from real patient CT data, 2) represent full DBS systems, and 3) are completely new (no overlap with those that were presented in Ref 12). The figure below shows the superposition of the evaluated trajectories from this present study (black lines) and trajectories assessed in Ref 12 (magenta lines), illustrating no redundancy in the trajectories evaluated. The reason we chose to perform experiments with a completely new set of trajectories as well as a different DBS device model was to provide **independent new data** to increase the confidence in the reproducibility of the observed effect and to minimize the chance that what we observed was due to some peculiarity in the DBS device or selected trajectories.

| Figure: Superposition of evaluated DBS lead trajectories from this study (black lines) and trajectories from Ref 12 (magenta lines). No overlap was present across the trajectories. |  |
| --- | --- |

Introduction & Methods: The authors compare simulation results of SAR with measured temperature rise. I think thermal simulations instead of SAR would provide much more insight and provide a better metric for comparison. Please comment.

**Answer:** Please note, the goal of the simulations was not to “compare” simulations with measurements. The simulations were performed only to compare how different lead geometries (i.e., straight vs helical with different pitches) behave in both MRI environments. In this sense, there is no comparison between SAR and temperature. SAR is compared with SAR under different scenarios.

On another note, it is true that the same SAR can result in different temperature rise **in different tissues.** That is, if the thermal properties of two tissues (such as thermal conductance and specific heat capacity) are different, then they will heat up to different degrees even when exposed to the same SAR. However, this is not the case we are concerned about here, as the tissue remains the same, and only the SAR value is changing. Thus, thermal simulations would still show the same trend.

Methods part 2.2, page 7: Why have you studied 27 trajectories that were contralateral to the IPG, but only 3 that were ipsilateral to the IPG? Three is a very, very low number, and this choice of different trajectories has not been explained. Even in terms of statistical evaluations a set of 5 cases is the absolute minimum. Please comment.

**Answer:** This is a great question. Our choice was based on the results of ~200 experiments that we had performed in a separate study (currently unpublished) comparing RF heating of ipsilateral and contralateral leads. We had compared measured temperature rise around the tip of 150 unique contralateral DBS trajectories with the temperature rise around tips of 94 unique ipsilateral trajectories and found that contralateral DBS leads generate substantially higher heating (see Figure below). This means that contralateral leads are the bottleneck of safety limits in horizontal systems, and that’s why our focus was on contralateral leads; however, we still examined a few ipsilateral cases just to make sure that they do not unexpectedly heat up in vertical MRI systems.

Figure: (a) Top view of superposition of evaluated DBS lead trajectories contralateral and ipsilateral to the IPG. (b) Superposition of DBS lead trajectories contralateral to the IPG (black). (C) Superposition of DBS lead trajectories ipsilateral to the IPG (red). (d) Distribution of the measured maximum temperature increase at the DBS lead-tip for trajectories contralateral and ipsilateral to the IPG. The maximum temperature increase was significantly lower for ipsilateral leads (p = 2.44 x 10^-23^ from two-sampled, one-tail t-test where statistical significance was established for p< 0.05). The mean ± standard deviation of the maximum temperature increase were 3.44 ± 1.93 ^o^C and 1.26 ± 1.17 ^o^C for lead trajectories contralateral and ipsilateral to the IPG, respectively.

Methods part 2.2, page 8: The temperature rise was measured for less than 4 minutes only! Why was the measurement time so short? When I look at the curves I do not so a flattening of the curves that would justify an early abortion of the measurement, but a continuous increase in temperature. Please comment.

**Answer:** The reviewer observation is correct, but please note that our goal here was not to determine the absolute maximum temperature rise in each case (for which we should have continued measurements until the steady-state flat curve was reached), but to compare RF heating of different cases. Especially, note that the temperature profiles in the vertical coil flatten much faster than that of the conventional coil (See Figure 4a). Thus, even if we had continued the measurements for longer, the results would have predicted the same trend.

The shorter measurement duration was a compromise between the number of unique trajectories that we could examine in the limited access time that we had to the scanner versus measuring for longer time but for fewer trajectories, and we decided that the former approach was more reasonable to do in this case.

Figure references on page 9 and 11: Figures 4 and 5 are mixed up. Please revise their order.

**Answer:** Thank you for the observation. The labeling has been corrected in the revised manuscript.

Methods page 9, Table 3: If I understand correctly, the authors addressed their second aim of the study, i.e. the transferability of the previous simulation results to leads from other manufacturers by three configurations only. A straight wire was compared to helical wires with pitches of 1 mm and 2 mm, but not for 30 different trajectories. Correct? Is that really enough evidence to generalize your results to DBS devices from other manufacturers? Please do not get me wrong. I understand that all the simulations are time-consuming and extensive, but I do see a mismatch in answering your two questions here. First, I see that already 90 lead trajectories have been compared between both scanner systems along with 12 selected temperature measurements in the 2021 MRM publication, and still, you add another 30 measurements in this study. On the other hand, for the second question 3 simulations without supporting experimental measurements should be sufficient.

**Answer:** Regarding the number of experimental studies between 2021 MRM paper and the current paper, please note that there were **only 4 trajectories tested with a commercial DBS device** in the MRM paper. The other 8 experiments were performed with **insulated wires**, not DBS systems, and with the same 4 trajectories (that is, there were not 12 unique trajectories). Moreover, the trajectories of the lead were all artificial and not based on patient data. So, the comparison is between 30 unique patient derived trajectories tested on a real DBS system (here) and 4 artificial trajectories (see Figure 6c of MRM paper) tested with **another DBS system** (MRM 2021 paper).

Regarding the simulations, the majority of the 90 simulations in the MRM 2021 paper were **lead only** systems and all with a **straight core** (i.e., only a 40-cm lead was modeled, not the extension cable or the IPG). The addition of the helical core substantially increases the simulation time as it significantly increases the complexity of the mesh. For example, the simulation time for a typical simulation in MRM 2021 paper was only 240 minutes, while the simulation time for our full-device helical lead here was 1854 minutes. For this reason, it is practically impossible for us to perform simulations for all 30 trajectories with helical leads. That being said, we agree with the reviewer that more data is needed to increase the confidence in the results. Therefore, we have added 4 additional simulations with helical leads and 2 additional simulations with straight leads following trajectories that represented the median and small differences in the measured maximum temperature increase between the two MRI systems.

Results, which showed the same trend as reported before, are now reflected in the revised text and Figure 6.

Reviewer #2: This well-written manuscript investigates the possibility of safe MRI scanning the patients with DBS lead in vertical, open-bore scanners. The first objective of the authors is to explore the validity of their previously-present numerical results using a vertical scanner and a commercial DBS lead. Their second objective is to extend the outcomes of this work to different types of commercial DBS leads from other vendors. Their experimental results show more than ten folds reduction in maximum temperature rise if a vertical scanner is used instead of a horizontal scanner. Although I believe this work contains critical scientific and practical information that may significantly impact the pathway toward DBS patients’ imaging, two major concerns and several minor issues need to be addressed before publication.
Major:
1. One of the main objectives of this manuscript is to extend the presented experimental results to other commercial DBS leads. To accomplish this objective, numerical simulations of a DBS lead with different internal wiring structures were exploited. However, only one trajectory (not to mention that the trajectory generates a large difference in Tmax between RF exposures in the horizontal versus vertical scanners) was used for this purpose. To be inclusive, please perform a similar analysis for all trajectories (30 available trajectories). Presenting a graphic similar to Fig. 4C (30 trajectories, each containing 6 bars: 2 Scanners × 3 Wiring Structures) would be ideal.

**Answer:** Please see our answer to the question by reviewer 1. Considering the substantial increase in the computation time due to the addition of the helical structure, it is practically impossible for us for perform simulations on all 30 trajectories (we simply do not have enough computational resources for this). However, we agree with the reviewer that more data is needed to increase the confidence in the results. Therefore, we have added 4 additional simulations with helical leads following trajectories that represented the median and small differences in the measured maximum temperature increase between the two MRI systems.

Results, which showed the same trend as reported before, are now reflected in the revised text and Figure 6.

2. In heating studies (TSE sequence), the scanner-reported B1rms value is chosen as the control metric and set to a fixed value for all cases (4uT). To the best of the reviewer’s knowledge (please explain if it is otherwise), B1rms is a pre-determined value by the scanner’s vendor based on numerical simulations with the corresponding coil model. If this is the case, the reported value does not represent a fair comparison criterion for different scanners from different vendors.

**Answer:** This is a good point and a common misconception that we wish to clarify here:

When performing an MRI exam on patients implanted with an MR Conditional active implant and/or device, it is often critical to be aware of, and control the amount of RF power utilized. Historically, the metric utilized for this purpose has been SAR. **However, SAR has been shown to be an inaccurate metric for RF-induced heating relating to implants and devices** (please see IEC 60601-2-33:2010+A11:2011 and Corrigendum 1:2012. Medical electrical equipment. Part 2-33: Particular requirements for the basic safety and essential performance of magnetic resonance equipment for medical diagnosis. 3rd. Geneva: International Electrotechnical Commission, 2010).

No universal standard is in place for estimating SAR. Therefore, the method, calculations and resultant value reported **will vary with the MR equipment manufacturer**. Each manufacturer uses their own assumptions regarding the patient’s size and tissue composition to estimate the SAR. Based on these known factors, regarding SAR estimation, we expect that displayed SAR values will vary greatly depending on the patient and the MR equipment vendor. To address this historic problem with SAR, a Joint Working Group, comprised of engineers and scientists from MR equipment and device manufacturers along with the FDA, has recommended B1+rms be used as a metric for device heating as opposed to SAR, **and in 2013, the IEC (International Electrotechnical Commission) mandated all MR systems manufactured going forward must display the B1+rms.**

The term *B1+rms* refers to the "root mean square" value of *B1+* averaged **over a period of 10 seconds** (see figure below).

The **IEC** describes a method by which manufacturers may calculate *B1+* using the principle that *B1+* scales linearly with the voltage supplied to the transmitter. However, because each patient presents a different electronic load to the RF coil, the voltage required to attain a given flip angle must be empirically determined. This calibration occurs during pre-scan, where 1 ms rectangular pulses of different amplitude are tested until the reference transmitter voltage (*Vref*) to achieve a perfect 180º-pulse is determined. Once *Vref* is known, the magnitude of the *B1+* field for any other transmitter voltages can be easily calculated.

An important characteristic of B1+rms is that it is **not an estimated value** but a **known value based on the pulse sequence and the associated parameters**. It is not patient dependent nor is it calculated differently based on the MR system manufacturer. A given B1+rms value on system A is the same on system B.

Minor:
3. Introduction (P3.Para3): Please include a brief description of RMS B1+

**Answer:** Thank you for the comment. We have included a brief description of RMS B1+ in the introduction of the revised manuscript.

4. Introduction (P4.Para2): “… which produces a notably different field distribution …” please change the “field” to either “EM field” or “electric field”. Please also add a proper reference for this statement (e.g., your previous publication: doi.org/10.1002/mrm.28818).

**Answer:** “Field” has been clarified to “EM field” in the revised text.

5. Materials and Methods (P5.Para4): Is the dataset of segmented models (30 realistic trajectories) publicly available?

**Answer:** Our goal is to ensure that our results are reproducible, and that the scientific community can build upon our work. For this reason, we will share all 3D models of body and implants upon written request to the corresponding author. The reason we have not uploaded this data to an online repository is that different users would need the data in various formats.

However, we have provided the DBS lead trajectories that were evaluated in this study in S2 File.

6. Materials and Methods (P6.Para2): Please also add the relative permittivity of the remaining head-torso phantom.

**Answer:** Thank you for this observation. The relative permittivity value for the saline in the head-torso phantom has been added.

7. Materials and Methods (P6.Para3): “… heterogenous human body model …”. Is it the DUKE model? Please add a proper reference.

**Answer:** The heterogenous human body model used in the simulations comparing the electric field distributions of the human body model and the experimental phantom was an ANSYS standard model. This is described in the text in S1 File.

8. Materials and Methods (P6.Para3): “… Supplementary Figures S3 and S4…”. Although it is presented as a good agreement, some noticeable discrepancies are visible. Since the hot spot ring is fully closed in the phantom, my first guess is a higher permittivity in the phantom (shorter wavelength). Please comment on this.

**Answer:** This is a good observation. However, it is important to note that the anthropomorphic phantom only included three tissue classes while the heterogenous human model consisted of 32 tissue classes. The hot spot ring coincided with the material of the 3D-printed skull (not the agar that filled the cavity inside the skull phantom). The skull phantom has some thickness to it, which can be clearly seen when looking at the cross-section of the skull phantom. In the simulation with the anthropomorphic phantom, the materials used—and the corresponding conductivity and permittivity values—reflect the materials of the experimental phantom (ABS plastic for the 3D-printed skull, agar for the brain, and saline for the head and torso). Given the selection of materials, the regions inside and outside the skull have electric field distributions that are similar to the distributions inside the human body model.

9. Materials and Methods (P7.Para2): I understand that two temperature probes were attached to the lead. However, only one temperature rise is given for each case in Fig. 4A. What is the use of the other probe?

**Answer:** This is correct; there were two temperature probes in case one of them faulted during measurements. We were only interested in the maximum temperature increase values measured out of both probes which are the values shown in Figure 5.

10. Materials and Methods (P7.Para2): Is the IPG set to the MR-mode as instructed by the vendor (Abbott)? If not, please investigate at least one case in MR-mode (both scanners).

**Answer:** The device was off in our case which is now mentioned in the revised text. However, we performed additional experiments with the device on and set to MRI mode and observed no difference in the heating. Results of those experiments are presented below.

Figure: (left) Clinician Programmer from Abbott for their DBS systems. (middle) Device is on and set to MRI-mode. (right) Device with stimulation off.

Figure: Maximum temperature increase of different trajectories from the additional experiments. No differences were found between the temperature increases when the device was in MRI-mode versus when the device was turned off.

11. Materials and Methods (P9.Para2): Fig. 4A and 4B are mislabeled ◊ Fig. 5A and 5B

**Answer:** Thank you for the observation. The labeling has been corrected in the revised manuscript.

12. Materials and Methods (P9.Para2): “… mean B1+ of 4uT on a central transverse …”. Is that adjusted to be 4uT in the presence or absence of the lead?

**Answer:** The mean B_1+_ of 4 $\mu$T was adjusted in the presence of the lead, but the central transverse plane did not intersect the lead or any component of the DBS implant.

13. Materials and Methods (P9.Para3); Equation 1: Please replace x with bar{r}. It can easily be misinterpreted as the x-coordinate of the cartesian coordinates system.

**Answer:** The x has been replaced with bar{r} in Equations 1 and 2.

14. Materials and Methods (P10.Para1); Equation 2: There are some issues/errors in this definition as well as its utility:
a. The result of the given integration has a time dependence. However, peak-to-peak voltage is usually defined as a constant value between the min and max of a monochromatic signal.
b. Since the voltage is defined as the difference in the electric potential between two points, the corresponding points need to be explicitly mentioned. If presumably, the integration is along the lead, then the defined voltage is between two ends of the DBS lead. However, the quasi-static assumption does not hold for such an elongated lead, and the “induced EMF” loses its meaning. Since this voltage is presented as a metric to compare the RF heating in different cases, please either justify it or change the metric (e.g., induced current, pSAR, etc).

**Answer:** (a) Equation 1 is a function of time. The integration in equation 2 is a function of time as well; however, the vertical bar in front of the integral (with the superscript “peak to peak” indicates that the Vpp is calculated as the difference between the maximum and the minimum of the time function over one period, and thus, is a number as expected. Figure 4 clearly shows this: the integral in Equation 2 is varying over time; however, Vpp is calculated as the peak-to-peak value of this time-varying function.

(b) The integration was originally taken along the total length of the lead. The wavelength of the RF field in the tissue at 1.5 T is ~60 cm, and it’s true that we are approaching the non-quasi static regime. We have recalculated the Vpp over the first 10-cm extracranial portion of the lead (integration start and end points P and Q are now illustrated in Figure 4).

15. Results (P11.Para1): Fig 4 ◊ Please correct the order of Fig. 4 and 5

**Answer:** Thank you for the observation. The labeling has been corrected in the revised manuscript.

16. Fig 5: Is the shield of the birdcage coil included in simulations? Is the vertical coil shielded?

**Answer:** Yes, the shield was included in both the birdcage and vertical coils. The diagrams of the coil in Figures 4a and 4b have been revised to include labeling of the shields.

17. Results (P11.Para2): Fig 5:
a. Is the vector presentation corresponding to the incident E-field? Please mention it.
b. The simulation software computes the total E-field (incident + scattered). How do you distinguish the incident field?
c. Is the color presentation over the lead corresponding to the tangential E-field? Please mention it.

Answer: (a) Yes, the vector presentation corresponds to the incident E-field. This is mentioned in the figure caption.

(b) As mentioned in the Methods, separate simulations were performed to determine the incident electric field and SAR. In order to calculate the incident electric field, simulations were performed with only the human body model; the implant was not included in the simulation. Non-model polylines representing the trajectory were imported into the simulations during post-processing to quantify the distribution of the tangential component of the incident electric field.

(c) Yes, the colormaps show the tangential E-field. This has been updated in the revised figure caption.

18. Discussion (P12.Para3): “… conventional solenoidal birdcage coils …” Birdcage coils are not solenoidal structures. Please remove the term.

**Answer:** Thank you for the comment. The term solenoidal has been removed.

19. Discussion: Although it is an interesting idea to use a helical shape to mimic the internal wiring structure of DBS leads, it is not accurate modeling, especially for multichannel leads (with complex wiring strategies). Please discuss this matter.

**Answer:** While we understand that the helical wire structures that we modeled still deviate from the structure of actual DBS leads, the goal of the simulations with different types of internal wire structures was to better understand RF heating of DBS leads with different electrical lengths in both types of MR environments. Previous simulations were performed with simplified straight internal wires to represent DBS leads. However, such simulations assume that 1) actual DBS leads do not have a ribbon-like internal structures and that 2) the electrical lengths of DBS leads are the same when their apparent lengths are the same. The goal of the simulations was to show that vertical MRI systems can substantially reduce RF heating for DBS leads that have different internal structures (which is the likely case for DBS leads from different manufacturers) and thus different electrical lengths despite having the same physical lengths.
